# Cannabinoids in Alzheimer’s disease: animal–human evidence and clinical pharmacology challenges

**DOI:** 10.3389/fnbeh.2026.1833021

**Published:** 2026-05-13

**Authors:** Maria Komedera, Urszula Wojda, Anna Kiryk

**Affiliations:** Laboratory of Preclinical Testing of Higher Standard, Nencki Institute of Experimental Biology, Polish Academy of Sciences, Warszawa, Poland

**Keywords:** Alzheimer’s disease, cannabinoids, endocannabinoid system, patients, transgenic mouse models

## Abstract

Cannabinoids have emerged as potential modulators of pathological processes in Alzheimer’s disease (AD), including neuroinflammation, synaptic dysfunction, and protein aggregation. Cannabidiol (CBD) and Δ9-tetrahydrocannabinol (THC), the main phytocannabinoids from *Cannabis sativa*, interact with the endocannabinoid system and may influence neuronal and glial signaling pathways relevant to AD pathology. This mini review summarizes evidence from transgenic animal models and clinical studies evaluating CBD, THC, and their combination in AD. Preclinical studies show that CBD and THC reduce *β*-amyloid accumulation, attenuate tau phosphorylation, and regulate neuroinflammatory responses, often associated with improvements in learning and memory. Cognitive outcomes appear to depend on cannabinoid composition, with CBD or THC administered individually showing more consistent effects, while combined CBD + THC effects appear dose- and ratio-dependent. Clinical evidence in AD patients remains limited and primarily reports improvements in neuropsychiatric symptoms, such as reductions in agitation, nighttime activity, and behavioral disturbances, whereas cognitive improvements are modest. Cannabinoid-based treatments are generally well tolerated, with mild sedation, somnolence, or disorientation as the most reported adverse effects. Overall, current data support the biological plausibility of cannabinoids as modulators of neuroinflammatory and synaptic processes in AD. However, heterogeneity in formulations, dosing, and study design limits firm conclusions. Future research should focus on dose optimization, biomarker-guided clinical trials, and long-term safety assessments to better define their therapeutic potential in AD.

## 1 Introduction

Interest in the potential therapeutic role of cannabinoids in Alzheimer’s disease (AD) has increased in recent decades, although the medicinal use of cannabis dates back centuries in traditional medicine, and systematic pharmacological studies on cannabinoids began in the mid-20th century. Cannabis has historically been used primarily for recreational purposes, but more recently also for medicinal purposes in individual AD patients presenting symptoms such as food refusal (Volicer et al., 1997), aggressiveness and agitation (Passmore, 2008), or nighttime agitation (Walther et al., 2006). Although these reports described positive behavioral outcomes, no inflammatory, neurodegenerative, or cognitive markers were assessed. Moreover, the Specialized Register of the Cochrane Dementia and Cognitive Improvement Group concluded that there was no evidence to support the effectiveness of cannabinoids in improving behavioral symptoms or other clinical outcomes in dementia, highlighting the need for well-designed randomized controlled trials.

Pharmacological studies in animals, initiated in the 1940s with individual cannabinoids, led to the identification of the two main active cannabinoids, cannabidiol (CBD) and tetrahydrocannabinol (THC), which interact with cannabinoid CB1 and CB2 receptors. These findings contributed to the characterization of the endogenous cannabinoid system (ECS), which is now recognized as an important regulatory system involved in both physiological and pathological processes. Consequently, drugs capable of mimicking, enhancing, or blocking the actions of endogenously released cannabinoids were proposed as potential therapeutic agents (Pertwee, 2006). THC and CBD are the most extensively characterized phytocannabinoids, supported by a substantial body of *in vivo* and clinical evidence demonstrating their pharmacological relevance. Beyond CB1- and CB2-dependent signaling, both compounds engage multiple non-CB receptor dependent mechanisms, including modulation of ion channel activity, redox homeostasis, mitochondrial function, and intracellular signaling cascades, which collectively contribute to their pleiotropic neurobiological effects.

Interest in cannabinoids subsequently led to the development and approval of synthetic analogs of Δ9-THC, such as dronabinol and nabilone, in the 1980s for the treatment of chemotherapy-induced nausea and vomiting. Later, the first cannabis-derived medicinal product containing both Δ9-THC and CBD, Sativex® (nabiximols), was licensed in Canada in 2005 as an adjunctive treatment for the symptomatic relief of neuropathic pain in adults with multiple sclerosis (Robson, 2005).

The next step was to target the ECS as a potential therapeutic approach for AD, particularly in its early stages. The first clinical studies conducted between 1997 and 2011 evaluated dronabinol and nabilone in small cohorts of AD patients, primarily assessing their effects on neuropsychiatric and behavioral symptoms of dementia.

One of the early preclinical studies demonstrated that intracerebroventricular administration of the synthetic cannabinoid receptor agonist WIN55,212–2 in rats prevented *β*-amyloid-induced microglial activation, cognitive deficits, and the loss of neuronal markers, suggesting a neuroprotective role of cannabinoid receptor activation in experimental models of AD (Ramírez et al., 2005). Subsequently, with the development of transgenic models of AD, it became possible to investigate the short- and long-term multifactorial effects of cannabinoids on immunological, behavioral, and cognitive functions.

This review focuses on evidence derived from transgenic models of Alzheimer’s disease and clinical studies conducted in patients with AD treated with CBD, THC, or their combination. The scope was restricted to THC and CBD because these compounds, and related THC/CBD-based formulations, currently have the strongest *in vivo* and clinical evidence base in AD and the best-characterized mechanisms of action, including CB1-, CB2-, and receptor-independent pathways. Synthetic cannabinoid-based interventions directly related to this focus, such as dronabinol and nabilone, were retained because of their pharmacological relationship to THC. Other phytocannabinoids, including non-psychoactive compounds, as well as broader classes of synthetic cannabinoids, represent potentially relevant areas of investigation; however, the AD-specific evidence for these agents remains limited, fragmented, and heterogeneous, which makes a balanced comparative synthesis difficult.

## 2 Preclinical and clinical outcomes

### 2.1 Evidence from animal models

Studies of animal models suggest that two main cannabinoids may influence multiple pathological pathways in AD, including amyloid and tau pathology, neuroinflammation, and behavioral and cognitive dysfunction. The magnitude and direction of these effects depend strongly on the cannabinoid compound, administered dose, animal model, age, and sex.

#### 2.1.1 Cannabinoid effects on AD pathology and cellular mechanisms

Across multiple AD animal models, cannabinoids influence several key pathological hallmarks of the disease. Administration of CBD or THC has been shown to reduce *β*-amyloid accumulation and modulate plaque dynamics in transgenic AD models such as APP/PS1 and 5xFAD mice, following systemic (oral, intraperitoneal) or intranasal administration at low, moderate, or high doses and over short (≤2 weeks), intermediate (2–8 weeks), and long-term (>2 months) treatment durations. Minimal doses at which reductions in amyloid pathology were observed included CBD (5 mg/kg delivered systemically for 30 days in APP/PS1 mice), accompanied by decreased hippocampal Aβ levels and RNA-seq analysis revealing differential expression of genes involved in immune and phagosome pathways, including Tlr2, Ccl2, Cd68 and Fcgr3, as well as autophagy-related genes such as Becn1 and Atg5 (Hao and Feng, 2021). For THC, reductions in amyloid pathology were reported even at ultra-low doses, including 0.002 mg/kg administered intranasally for 3 months in APP/PS1 mice, where the effect was accompanied by decreased Aβ1-40 and Aβ1-42 levels and reduced levels of phosphorylated tau (Fihurka et al., 2022). Intranasal delivery may partially bypass the blood–brain barrier (BBB), enabling higher local brain concentrations of THC which, together with the high sensitivity of the endocannabinoid system and activation of neuroprotective signaling pathways, may explain the hormetic responses observed at ultra-low cannabinoid doses (Nitzan et al., 2022; Fihurka et al., 2022). Systemically administered THC also reduced amyloid-related pathology at doses as low as 0.02 mg/kg delivered intraperitoneally for 3 months in APP/PS1 mice, accompanied by reduced Aβ oligomers, decreased tau phosphorylation, and lower activity of glycogen synthase kinase-3β (GSK-3β), a kinase implicated in tau pathology (Wang et al., 2022).

Reductions in phosphorylated tau levels have also been reported, ability to target multiple pathological processes simultaneously at the same dosage, whereas similar THC doses were effective at comparable treatment durations to those affecting amyloid pathology. Minimal doses associated with attenuation of tau pathology included CBD (10 mg/kg administered orally for 4 weeks in 5xFAD mice) and THC (0.02–0.2 mg/kg delivered intraperitoneally for 3 months in APP/PS1 mice) (Raïch et al., 2025; Wang et al., 2022). In the case of CBD, reductions in tau pathology occurred together with changes in glial activation and neuroinflammatory signaling, including decreased astrocyte activation and altered microglial responses (Raïch et al., 2025). By contrast, attenuation of tau pathology following THC treatment was accompanied by reduced activity of GSK-3β (Wang et al., 2022).

Combined administration of CBD and THC has also been investigated in several AD models, although the effects appear to depend strongly on dose and cannabinoid ratio. Low-dose combinations administered systemically have been reported to modulate amyloid pathology and improve cognitive performance. For example, intraperitoneal administration of CBD (0.273 mg/kg) and THC (0.205 mg/kg) for 28 days in 5xFAD mice improved spatial memory and altered plaque composition, increasing insoluble Aβ and suggesting sequestration of toxic soluble amyloid species (Arnanz et al., 2024). Similarly, combined treatment with CBD and THC (0.5 mg/kg each for 5 weeks) in APP/PS1 mice reduced soluble Aβ42 levels and modified plaque characteristics, accompanied by decreased extracellular glutamate and increased astrocytic glutamate uptake (Sánchez-Fernández et al., 2024). However, higher doses or imbalanced cannabinoid ratios did not consistently produce beneficial outcomes. In APP/PS1 mice, treatment with THC (3 mg/kg) combined with CBD (20 mg/kg) failed to rescue cognitive deficits and increased anxiety-like behavior (Aumer et al., 2025). Likewise, very high CBD-dominant combinations (CBD: THC 99:1, 50 mg/kg) in 5xFAD mice increased anxiety- and depressive-like behaviors despite reducing plaque complexity and enhancing microglial phagocytosis (De Martín Esteban et al., 2026). Together, these findings suggest that while combined cannabinoid administration can influence amyloid pathology, its therapeutic potential in AD models is highly dependent on dose, treatment duration, and cannabinoid ratio.

#### 2.1.2 Behavioral and cognitive effects of cannabinoids

Cannabinoid treatment in AD models was occasionally associated with changes in general behavioral activity. High-exposure cannabinoid regimens were linked to reduced locomotion and altered behavioral engagement. For example, twice daily subcutaneous administration of a high-dose CBD: THC 99:1 mixture (50 mg/kg) for 28 days reduced locomotor activity and increased anxiety- and depression-like behavior in male 5xFAD mice, despite evidence of enhanced microglial phagocytosis and reduced amyloid accumulation in neuritic plaques (de Martín Esteban et al., 2026). These findings suggest that behavioral worsening is more likely under conditions of high cumulative exposure or unfavorable CBD: THC ratios rather than representing a uniform class effect.

Cannabinoid administration also influenced emotional reactivity. Chronic CBD treatment reduced anxiety-like behavior and contextual fear-associated freezing in 14-month-old female TAU58/2 mice (Kreilaus et al., 2022). In contrast, higher exposure or imbalanced cannabinoid regimens were associated with increased anxiety- or depressive-like behavior, suggesting that anti-amyloid effects at high exposure do not necessarily translate into favorable emotional outcomes and may fall outside the optimal therapeutic window (Aumer et al., 2025; de Martín Esteban et al., 2026).

The most consistently reported behavioral effects of cannabinoids in AD models concerned cognitive performance. Across multiple studies, cannabinoid treatment improved spatial learning, working memory, and object recognition, and in some cases stabilized or prevented cognitive decline in transgenic AD models. These benefits were observed across different stages of disease progression, including early-stage APP/PS1 mice (6 months) after combined THC + CBD treatment (Sánchez-Fernández et al., 2024), intermediate-stage 5xFAD mice (8 months) treated with low-dose CBD + THC combinations (Arnanz et al., 2024), and 12-month-old APP/PS1 mice receiving long-term intranasal THC (Fihurka et al., 2022). Cognitive improvements were also observed in advanced stages, including 14-month-old TAU58/2 mice after chronic CBD administration and APP/PS1 mice aged 14–17 months after low-dose systemic THC (Wang et al., 2022; Kreilaus et al., 2022).

These findings suggest the presence of a dose-dependent therapeutic window in which appropriately balanced cannabinoid treatments may confer cognitive benefits, whereas excessive exposure or unfavorable cannabinoid ratios may shift outcomes toward behavioral dysregulation.

### 2.2 Evidence from human studies

Clinical studies evaluating cannabinoids in patients with AD primarily report effects on three domains: cognitive function, neuropsychiatric symptoms, and treatment tolerability. Overall, available evidence suggests that cannabinoids exert more consistent effects on behavioral and neuropsychiatric symptoms than on cognitive decline in AD (Table 1).

**Table 1:** Cannabinoid interventions in Alzheimer’s disease: evidence from animal models and clinical studies.

Study	Model (n)/AD patients (n)	Age/Sex	Cannabinoid/administration	Dose/Duration	Cognitive outcomes	Amyloid/Tau effects	Proposed mechanism
Transgenic mouse models of Alzheimer’s disease							
Arnanz et al. (2024)	5xFAD (16)	8 months male	CBD + THC i.p.	CBD 0.273 mg/kg + THC 0.205 mg/kg, 28 days	Improved spatial memory	↑ insoluble Aβ	Possible sequestration of toxic Aβ oligomers
Aumer et al. (2025)	APP/PS1 (10)	14.5 months female	THC + CBD i.p.	THC 3 mg/kg + CBD 20 mg/kg, 3 weeks	No cognitive rescue; ↑ anxiety	Not assessed	Cannabinoid interaction affecting behavior
Cheng et al. (2014)	APP/PS1 (23)	24–32 weeks male	CBD i.p. injection	20 mg/kg, 8 weeks	Restored object recognition and social memory	Not assessed	Not assessed
Chesworth et al. (2022)	APP/PS1 (12)	2.5 months female	CBD oral gel pellets	20 mg/kg daily, 8 months	Moderate improvement in spatial learning	Not assessed	Possible neuroprotection independent of amyloid
De Martín Esteban et al. (2026)	5xFAD (31)	3, 6, 9 months male	CBD: THC 99:1 s.c.	50 mg/kg or twice daily, 28 days	↑ anxiety & depression-like behavior; memory unchanged	↓ plaque complexity	↑ microglial phagocytosis
Dosseto et al. (2025)	APP/PS1 (7)	Female, 10 months	CBD i.p. injection	20 mg/kg, 2 months	Not assessed	Not assessed	No effect on copper homeostasis; Zn and Fe unchanged
Fihurka et al. (2022)	APP/PS1 (10)	12 months	THC intranasal	0.002–0.02 mg/kg, 3 months	Prevented spatial memory decline	↓ Aβ1-40, Aβ1-42; ↓ p-Tau	Enhanced Aβ clearance
Goodland et al. (2025)	SAMP8 (6–8)	11 months male	CBD oral gavage	3–30 mg/kg, 2 months	Improved learning and memory	Not assessed	↓ oxidative stress; ↑ mitochondrial function
Hao and Feng (2021)	APP/PS1 (6)	6 months male	CBD i.p. injection	5 mg/kg, 30 days	Improved spatial memory	↓ Aβ plaques; ↑ autophagy	↑ immune/phagosome pathways
Jin et al. (2025)	5xFAD (6–8)	2 months male	CBD i.p. injection	5 mg/kg, 30 days	Improved cognition	↓ tau aggregation	Autophagy induction
Khodadadi et al. (2021)	5xFAD (6–10)	9–12 months male	CBD i.p. injection	10 mg/kg every other day, 2 weeks	Improved cognition	↓ Aβ accumulation	↑ TREM2 & IL-33 → enhanced microglial phagocytosis; ↓ IL-6
Khodadadi et al. (2024)	5xFAD (10)	5 months male	CBD inhalation	10 mg/mouse, 7 months	Improved cognition	↓ amyloid pathology	↑ acetylcholine signaling; immune modulation
Kreilaus et al. (2022)	TAU58/2 (9)	14 months female	CBD i.p. injection	100 mg/kg, 7 weeks	Restored spatial memory	Tau inhibition suggested	↑ hippocampal neurogenesis; ↑ BDNF; anxiolytic-like effect
Naeini et al. (2025)	5xFAD (10)	9–12 months male	CBD inhalation	10 mg/mouse, 4 weeks	Improved memory	↓ amyloid pathology	Modulation of IDO/cGAS; ↑ microglial activation; ↓ astrocytic activation; ↑ IL-10, ↓ IFN-γ, IL-1β, TNF-α
Nitzan et al. (2022)	5xFAD (8–10)	6 months female; 12 months female/male	THC injection	0.002 mg/kg single dose	Improved spatial and working memory	Not assessed	↑ TrkB signaling; ↓ inflammatory genes
Nitzan et al. (2026)	5xFAD (8)	3 months / both	THC injections	0.002 mg/kg (3 injections), 2 months	Improved cognition	Not assessed	↓ neuroinflammation (hippocampus in males, PFC in females)
Raïch et al. (2025)	5xFAD (8)	4 months male	CBD oral gavage	10 mg/kg, 4 weeks	Improved short- and long-term memory	↓ Aβ aggregation; ↓ p-Tau transport	↑ M2 microglia, ↓ astroglia, ↓ IL-1β, ↑ IL-10
Sánchez-Fernández et al. (2024)	APP/PS1 (7)	6 months/both	Injection	0.5 mg/kg each, 5 weeks	Potential cognitive improvement	↓ soluble Aβ42; altered plaques	↓ extracellular glutamate; ↓ hippocampal hyperexcitability; ↑ astrocytic glutamate uptake
Wang et al. (2022)	APP/PS1	14–17 months/both	THC i.p. injection	0.02–0.2 mg/kg, 3 months	Restored spatial learning	↓ Aβ oligomers; ↓ tau phosphorylation	↓ GSK-3β activity
Xiao et al. (2021)	APP/PS1 (3)	3 months male	THC intragastric	400 mg/kg/day, 5 months	Improved memory	↓ Aβ aggregation	Anti-inflammatory effects via microglial M2 activation and Ras/ERK signaling
Yang et al. (2024)	APP/PS1 (5–10)	6 months male	CBD i.p. injection	10 mg/kg, 1 week versus 21 days	Not assessed	Short-term: ↓ neuroinflammation; long-term: ↑ APP, ↑ Aβ plaques	TRPV2 phosphorylation → ↑ channel sensitivity ↑ microglial Aβ phagocytosis
Patients with Alzheimer’s disease							
Albertyn et al. (2025) Randomized controlled trial	AD patients (15)	~83 yrs	Nabiximols oral mucosa vs. placebo	1 → 4 sprays/day (2.7 mg THC + 2.5 mg CBD per spray), 4-week treatment + 4-week follow-up	↓ CMAI scores/↓ agitation	Not assessed	High feasibility; 100% retention and adherence; safe; no treatment-related adverse events
Cury et al. (2025) Clinical trial	AD patients (28)	60–80 yrs	Oral cannabis extract (THC + CBD)	THC 0.350 mg + CBD 0.245 mg daily, 26 weeks	Cognitive stabilization	Not assessed	Disorientation, headache, paranoia, somnolence, dry mouth, weight gain
Herrmann et al. (2019) Clinical trial	AD patients (38)	~87 yrs	Nabilone orally vs. placebo	1–2 mg/day, 6 weeks; 14-week crossover	Improved agitation, overall behavior, caregiver distress	Improvement in neuropsychiatric symptoms (NPI-NH)	↑ sedation
Rosenberg et al. (2026) Clinical trial	AD patients with agitation (75)	Severe cognitive impairment	Dronabinol vs. placebo orally	Titrated up to 10 mg/day, divided twice daily, 3 weeks	↓ agitation	Not assessed	Somnolence reported; no increased intoxication or major adverse events
Palmieri and Vadalà (2022) Clinical trial	AD patients (30)	65–90 yrs	Oil-diluted cannabis extract Bedrocan / sublingual oil, twice daily	22% THC (~220 mg/g) + 0.4–0.5% CBD (~4 mg/g), 12 weeks	↓ agitation, apathy, irritability, sleep & eating disturbances; ↓ caregiver distress	Not assessed	↓ aggressive behaviors
Walther et al. (2006) Open-label pilot	AD patients (5)	~81.5 yrs	Dronabinol capsules (Δ9-THC)	2.5 mg/day, 2 weeks	↓ nighttime motor activity and agitation	Not assessed	No adverse events reported

#### 2.2.1 Cognitive effects

Evidence for direct cognitive benefits remains limited. In a 26-week clinical trial, low-dose oral administration of THC (0.350 mg) combined with CBD (0.245 mg) was associated with stabilization of cognitive performance in patients with mild-to-moderate AD, although mild adverse effects such as somnolence, disorientation, and headache were reported (Cury et al., 2025). However, most other clinical studies primarily evaluated neuropsychiatric and behavioral symptoms rather than direct measures of cognitive decline.

#### 2.2.2 Behavioral and neuropsychiatric effects

THC-based or synthetic cannabinoids, including dronabinol and nabilone, reduced agitation, nighttime motor activity, and overall neuropsychiatric symptom burden in several studies (Walther et al., 2006; Herrmann et al., 2019; Rosenberg et al., 2026). Similarly, treatment with cannabis extracts containing high THC concentrations (Bedrocan oil) was associated with reductions in agitation, irritability, sleep disturbances, and eating disturbances, which in turn lowered caregiver distress (Palmieri and Vadalà, 2022). Balanced THC/CBD formulations such as nabiximols also demonstrated good feasibility and modest reductions in agitation in a randomized controlled trial (Albertyn et al., 2025).

#### 2.2.3 Safety and tolerability

Across studies, cannabinoid treatments were generally well tolerated in patients with AD. Plant-derived cannabinoid formulations, including oral cannabis extracts and balanced THC/CBD preparations such as nabiximols, were associated mainly with mild adverse effects including somnolence, headache, disorientation, and dry mouth (Cury et al., 2025; Albertyn et al., 2025). This more balanced tolerability profile may partly reflect the presence of cannabidiol (CBD), which can modulate some of the central effects of THC through interactions with the endocannabinoid system. Synthetic cannabinoids, including dronabinol and nabilone, more frequently produced sedation-related effects, likely reflecting their strong CB1 receptor agonist activity. For example, sedation was commonly reported during nabilone treatment (1–2 mg/day for 6 weeks) (Herrmann et al., 2019), while somnolence occurred in trials using dronabinol at doses titrated up to 10 mg/day (Rosenberg et al., 2026).

Importantly, serious adverse events or intoxication-like effects were rarely reported even at higher THC exposures, including dronabinol titrated up to 10 mg/day (Rosenberg et al., 2026) and plant-derived high THC cannabis extracts administered twice daily for 12 weeks (Palmieri and Vadalà, 2022).

Overall, clinical studies suggest that cannabinoids can help reduce agitation and behavioral symptoms in AD, with modest cognitive effects observed mainly for combined THC/CBD treatments and generally good tolerability.

## 3 Potential mechanisms and therapeutic window

**Figure 1:**
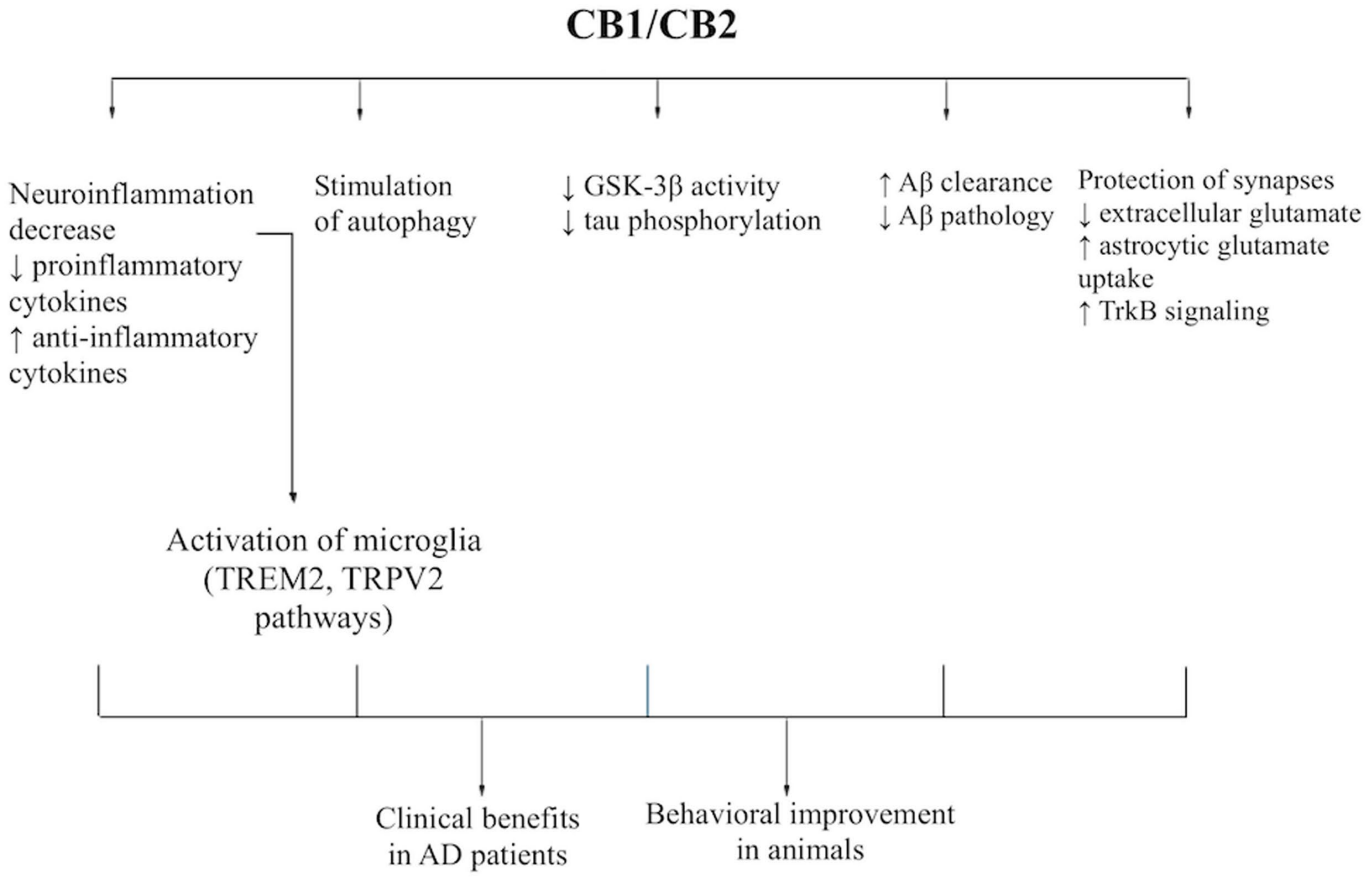
Proposed mechanisms of cannabinoid action in Alzheimer’s disease models. Cannabinoids may influence AD pathology through partially overlapping CB1- and CB2-dependent pathways. CB1 receptors, predominantly expressed in neurons, are linked to regulation of synaptic signaling, including modulation of glutamatergic transmission, astrocytic glutamate uptake, and TrkB-associated pathways. CB2 receptors, mainly expressed in microglia, modulate neuroinflammatory responses by decreasing pro-inflammatory cytokines (e.g., TNF-*α*, IL-1β, IFN-*γ*) and increasing anti-inflammatory signaling such as IL-10, while promoting microglial responses associated with amyloid clearance. These pathways may converge through autophagy- and phagosome-related mechanisms that facilitate the clearance of misfolded proteins. Downstream processes may include enhanced Aβ clearance and modulation of intracellular signaling pathways, such as reduced GSK-3β activity leading to decreased tau phosphorylation. Together, these mechanisms may contribute to improved cognitive and behavioral outcomes observed in experimental AD models and may translate into potential clinical benefits, including reduced agitation in patients with AD.

Alzheimer’s disease is driven by a network of pathological processes operating at both the intracellular and tissue /parenchymal levels. At the intracellular level, progressive amyloidogenic processing of amyloid precursor protein (APP), accumulation of toxic β-amyloid species, tau hyperphosphorylation, cytoskeletal destabilization, synaptic dysfunction, and impaired proteostatic clearance contribute to neuronal injury. At the tissue level, chronic neuroinflammation, glial dysregulation, and impaired clearance of toxic proteins from the brain parenchyma further promote amyloid and tau pathology, particularly in vulnerable regions such as the hippocampus. Against this background, cannabinoids may modulate several AD-relevant pathways through partially overlapping CB1- and CB2-dependent and receptor-independent mechanisms. However, not all proposed pathways are equally well documented, and some effects may involve additional molecular targets beyond the classical cannabinoid receptors (Figure 1).

### 3.1 Intracellular mechanisms relevant to AD pathology

A central intracellular contributor to AD pathogenesis is the shift of APP processing toward the amyloidogenic pathway, resulting in increased production and accumulation of toxic Aβ forms. These peptides disrupt neuronal homeostasis, impair synaptic signaling, and trigger downstream inflammatory and degenerative responses. Cannabinoids may alleviate this process indirectly by reducing Aβ burden, altering plaque composition, and enhancing cellular pathways involved in protein clearance. In experimental AD models, CBD, THC, and low-dose CBD + THC combinations have been associated with reduced Aβ accumulation or changes in amyloid pathology, suggesting that cannabinoid signaling may interfere with amyloid-related processes at multiple levels (Hao and Feng, 2021; Fihurka et al., 2022; Wang et al., 2022; Arnanz et al., 2024; Sánchez-Fernández et al., 2024).

Another major intracellular abnormality in AD is tau dysregulation. Hyperphosphorylation of tau, mediated in part by kinases such as glycogen synthase kinase-3β (GSK-3β), promotes tau detachment from microtubules, cytoskeletal destabilization, axonal dysfunction, and impaired synaptic communication. These changes contribute to neuronal dysfunction and the progression of tau pathology. Cannabinoids may partially counteract this process by reducing tau phosphorylation, including through mechanisms associated with lower GSK-3β activity, and by supporting cellular pathways involved in the clearance of abnormal tau forms (Wang et al., 2022; Raïch et al., 2025; Huang et al., 2023). In this way, cannabinoid-mediated modulation of tau-related signaling may help preserve neuronal integrity and limit progression of tau pathology, although the current evidence remains largely preclinical.

AD is also characterized by impaired intracellular proteostasis, including dysfunction of autophagy-related pathways responsible for the degradation of misfolded and toxic proteins. Reduced autophagic clearance may favor the intracellular accumulation of phosphorylated tau and toxic Aβ oligomers, thereby amplifying proteotoxic stress and neuronal dysfunction. Cannabinoids, particularly CBD in several experimental studies, have been linked to enhanced expression of autophagy-related genes and to mechanisms consistent with improved intracellular protein clearance (Hao and Feng, 2021; Jin et al., 2025). By supporting autophagic and proteostatic responses, cannabinoids may compensate for one of the key intracellular alterations in AD.

These intracellular abnormalities are closely linked to synaptic dysfunction, which is an early and functionally important feature of AD, especially in hippocampal circuits involved in learning and memory. Altered glutamatergic transmission and network instability contribute directly to cognitive decline. Through presynaptic CB1-related mechanisms and broader neuromodulatory actions, cannabinoids may reduce excessive neurotransmitter release, regulate extracellular glutamate, and stabilize synaptic activity. Such effects may help protect vulnerable synapses from excitotoxic damage and support cognition in AD models (Sánchez-Fernández et al., 2024; Nitzan et al., 2022; Jin et al., 2025).

### 3.2 Tissue- and parenchyma-level mechanisms in AD

At the tissue level, AD is marked by chronic neuroinflammation involving persistent activation of microglia and astrocytes. Activated microglia release pro-inflammatory cytokines such as TNF-*α*, IL-1β, and IL-6. Reactive astrocytes contribute to persistent inflammatory state and impaired support for neuronal homeostasis. Although glial activation may initially be protective, its chronic dysregulation promotes synaptic dysfunction, neuronal injury, and further accumulation of pathological proteins. Cannabinoids may mitigate this process by shifting glial responses toward a less inflammatory and potentially more protective phenotype, including reduced pro-inflammatory signaling and increased anti-inflammatory mediators such as IL-10 (Hao and Feng, 2021; Khodadadi et al., 2021; Naeini et al., 2025; Raïch et al., 2025). These effects are among the most consistently reported mechanisms in experimental AD models and may represent one of the main routes by which cannabinoids could slow disease progression.

In addition to intracellular clearance failure, AD also involves impaired elimination of toxic proteins from brain parenchyma. Reduced microglial clearance of extracellular Aβ aggregates, together with broader impairment of brain clearance systems, may favor the retention of amyloid species within vulnerable regions such as the hippocampus and cortex. In this context, cannabinoid effects on microglial phagocytosis appear particularly relevant. Available evidence suggests that cannabinoids may enhance phagocytic responses and modulate pathways associated with amyloid uptake and immune signaling, including TREM2- and TRPV2-related mechanisms (Khodadadi et al., 2021; Yang et al., 2024; Naeini et al., 2025; Jin et al., 2025). By improving glial handling of extracellular toxic proteins, cannabinoids may partly compensate for impaired parenchymal clearance in AD.

Other clearance pathways in the brain, including astrocyte- and blood–brain barrier-related mechanisms, also contribute to AD progression, but direct evidence for cannabinoid effects on these pathways remains limited.

These tissue-level abnormalities are particularly important in the hippocampus, where neuroinflammation, altered glutamate regulation, and impaired protein clearance converge with synaptic vulnerability to drive memory decline. Several cannabinoid effects described in experimental AD models, including modulation of glutamate dynamics, inflammatory signaling, and amyloid burden, have been reported in hippocampal tissue, supporting the idea that this region may be a key site of cannabinoid action in AD-related cognitive impairment (Hao and Feng, 2021; Sánchez-Fernández et al., 2024; Fihurka et al., 2022).

### 3.3 Cannabinoid receptor signaling

Activation of CB1 and CB2 receptors appears to initiate several of the cellular and tissue-level responses described above. CB1 signaling is particularly relevant to modulation of synaptic transmission and network activity, whereas CB2 signaling is more closely linked to microglial activation states, neuroinflammatory control, and phagocytic responses (Hao and Feng, 2021; Raïch et al., 2025; Naeini et al., 2025). At the same time, not all cannabinoid effects relevant to AD can be fully explained by classical CB1- and CB2-mediated signaling. Available evidence suggests that some actions may involve additional targets, including TRPV-related pathways, trophic signaling, and other receptor-independent effects (Nitzan et al., 2022; Yang et al., 2024; Jin et al., 2025). Therefore, cannabinoids should be viewed as pleiotropic modulators of AD-relevant processes rather than as agents acting through a single unified mechanism.

### 3.4 Therapeutic window and clinical implications

The therapeutic window refers to the dose range in which cannabinoids may exert beneficial effects, while lower doses may be subtherapeutic and higher doses may diminish benefit or increase adverse effects. This phenomenon is consistent with dose-dependent and sometimes biphasic effects of cannabinoid signaling, particularly through CB1 receptors. Low or ultra-low doses may preferentially activate neuroprotective signaling pathways, including TrkB-associated synaptic regulation and anti-inflammatory microglial responses, whereas higher doses can produce stronger CB1-mediated neuromodulatory effects that may lead to sedation or behavioral changes. This dose sensitivity may partly explain the therapeutic window observed in experimental models and suggested by clinical evidence.

Moreover, these mechanisms may differentially influence clinical domains in AD. While anti-inflammatory and synaptic effects may contribute to neuroprotection, modulation of neurotransmitter release and neuronal excitability may directly affect neuropsychiatric symptoms such as agitation and sleep disturbances.

## 4 Discussion

The available evidence indicates that cannabinoids influence several pathological and functional domains relevant to AD, although their clinical utility remains only partially defined. Preclinical studies consistently show that both CBD and THC can modulate key disease-related mechanisms, including *β*-amyloid accumulation, tau phosphorylation, neuroinflammation, and synaptic dysfunction. These effects appear to arise from partially overlapping CB1- and CB2-dependent pathways that regulate glial activation, neurotransmission, and cellular clearance mechanisms such as autophagy and microglial phagocytosis. In experimental models, these molecular effects are frequently accompanied by improvements in learning and memory.

However, translation of these findings to human studies remains limited. Clinical trials have primarily demonstrated benefits for neuropsychiatric symptoms, particularly agitation and sleep disturbances, whereas evidence for direct cognitive improvement is modest and inconsistent. A potential explanation is the presence of a dose-dependent therapeutic window. In several AD models, CBD and THC administered individually were associated with improvements in cognitive performance, while combined CBD + THC treatments showed stronger dependence on dose and ratio. Balanced low-dose combinations may produce beneficial effects, whereas higher exposure or unfavorable ratios can lead to behavioral dysregulation despite biochemical improvements.

Importantly, some cannabinoid neuroprotective effects may occur independently of classical CB1 and CB2 receptor signaling. Screening studies have shown that multiple cannabinoids can protect neurons from oxidative stress, intraneuronal Aβ toxicity, and loss of trophic support even in cells lacking CB1 and CB2 receptors. Structure–activity relationship analyses indicate that antioxidant functional groups, including aromatic hydroxyl moieties, contribute to these protective effects, suggesting that cannabinoid-derived compounds with combined receptor-mediated and intrinsic antioxidant properties may represent promising therapeutic candidates.

Nevertheless, several knowledge gaps remain. Dose mapping across studies is still limited, biomarker-based endpoints are rarely included in clinical trials, and long-term safety and potential drug interactions in elderly patients require further evaluation. Future research should prioritize long-term efficacy, optimal dosing strategies, and understanding underlying mechanisms to better define their therapeutic role. In addition, emerging evidence suggests that other phytocannabinoids, such as cannabidiolic acid (CBDA) and tetrahydrocannabinolic acid (THCA), may also exert anti-inflammatory and neuroprotective effects, highlighting the broader therapeutic potential of the cannabinoid family in AD (Kim et al., 2023).

In conclusion, cannabinoids represent a biologically plausible but still clinically underdefined therapeutic strategy in AD, with their future role likely depending on precise dose optimization, mechanistic understanding, and well-designed biomarker-guided clinical trials.

## References

[ref1] Albertyn C. P. Guu T.-W. Chu P. Creese B. Young A. Velayudhan L. . (2025). Sativex (nabiximols) for the treatment of Agitation & Aggression in Alzheimer’s dementia in UK nursing homes: a randomised, double-blind, placebo-controlled feasibility trial. Age Ageing 54:afaf149. doi: 10.1093/ageing/afaf149, 40479610 PMC12143470

[ref2] Arnanz M. A. Ruiz De Martín Esteban S. Martínez Relimpio A. M. Rimmerman N. Tweezer Zaks N. Grande M. T. . (2024). Effects of chronic, low-dose cannabinoids, cannabidiol, delta-9-tetrahydrocannabinol and a combination of both, on amyloid pathology in the 5xFAD mouse model of Alzheimer’s disease. Cannabis Cannabinoid Res. 9, 1312–1325. doi: 10.1089/can.2023.0101, 37862567

[ref3] Aumer B. Rosa Porto R. Coles M. Ulmer N. Watt G. Kielstein H. . (2025). Combination treatment with medium dose THC and CBD had no therapeutic effect in a transgenic mouse model for Alzheimer’s disease but affected other domains including anxiety-related behaviours and object recognition memory. Pharmacol. Biochem. Behav. 257:174101. doi: 10.1016/j.pbb.2025.174101, 40976394

[ref4] Cheng D. Low J. K. Logge W. Garner B. Karl T. (2014). Chronic cannabidiol treatment improves social and object recognition in double transgenic APPswe/PS1∆E9 mice. Psychopharmacology 231, 3009–3017. doi: 10.1007/s00213-014-3478-5 24577515

[ref5] Chesworth R. Cheng D. Staub C. Karl T. (2022). Effect of long-term cannabidiol on learning and anxiety in a female Alzheimer’s disease mouse model. Front. Pharmacol. 13:931384. doi: 10.3389/fphar.2022.931384, 36238565 PMC9551202

[ref6] Cury R. D. M. Da Silva T. Cezar-dos-Santos F. Fakih Y. R. C. Narvaez K. A. R. Gouvea M. C. . (2025). A randomized clinical trial of low-dose cannabis extract in Alzheimer’s disease. J. Alzheimer’s Dis 108, 1602–1613. doi: 10.1177/13872877251389608, 41160460

[ref7] Dosseto A. Tonge K. Karl T. (2025). Cannabidiol modulates brain copper homeostasis in wild-type-like but not Alzheimer’s disease transgenic female mice: implications for neuroprotective therapy. Mol. Neurobiol. 63:57. doi: 10.1007/s12035-025-05480-6, 41247623

[ref8] Fihurka O. Hong Y. Yan J. Brown B. Lin X. Shen N. . (2022). The memory benefit to aged APP/PS1 mice from long-term intranasal treatment of Low-dose THC. Int. J. Mol. Sci. 23:4253. doi: 10.3390/ijms23084253, 35457070 PMC9029288

[ref10] Goodland M. N. Banerjee S. Niehoff M. L. Young B. J. Macarthur H. Butler A. A. . (2025). Cannabidiol improves learning and memory deficits and alleviates anxiety in 12-month-old SAMP8 mice. PLoS One 20:e0296586. doi: 10.1371/journal.pone.0296586, 40811673 PMC12352845

[ref11] Hao F. Feng Y. (2021). Cannabidiol (CBD) enhanced the hippocampal immune response and autophagy of APP/PS1 Alzheimer’s mice uncovered by RNA-seq. Life Sci. 264:118624. doi: 10.1016/j.lfs.2020.118624, 33096116

[ref12] Herrmann N. Ruthirakuhan M. Gallagher D. Verhoeff N. P. L. G. Kiss A. Black S. E. . (2019). Randomized placebo-controlled trial of nabilone for agitation in Alzheimer’s disease. Am. J. Geriatr. Psychiatry 27, 1161–1173. doi: 10.1016/j.jagp.2019.05.002, 31182351

[ref13] Huang W. Huang J. Huang N. Luo Y. (2023). The role of TREM2 in Alzheimer’s disease: from the perspective of tau. Front. Cell Dev. Biol. 11:1280257. doi: 10.3389/fcell.2023.1280257, 38020891 PMC10663217

[ref14] Jin J. Fu C. Xia J. Luo H. Wang X. Chen S. . (2025). Cannabidiol ameliorates cognitive decline in 5×FAD mouse model of Alzheimer’s disease through potentiating the function of extrasynaptic glycine receptors. Mol. Psychiatry 30, 1817–1827. doi: 10.1038/s41380-024-02789-x, 39396064

[ref15] Khodadadi H. Salles É. L. Jarrahi A. Costigliola V. Khan M. Yu J. C. . (2021). Cannabidiol ameliorates cognitive function via regulation of IL-33 and TREM2 upregulation in a murine model of Alzheimer’s disease. J. Alzheimer’s Dis 80, 973–977. doi: 10.3233/JAD-210026, 33612548

[ref16] Khodadadi H. Salles É. L. Naeini S. E. Bhandari B. Rogers H. M. Gouron J. . (2024). Boosting acetylcholine signaling by cannabidiol in a murine model of Alzheimer’s disease. Int. J. Mol. Sci. 25:11764. doi: 10.3390/ijms252111764, 39519315 PMC11546302

[ref17] Kim J. Choi P. Park Y.-T. Kim T. Ham J. Kim J.-C. (2023). The cannabinoids, CBDA and THCA, rescue memory deficits and reduce amyloid-Beta and tau pathology in an Alzheimer’s disease-like mouse model. Int. J. Mol. Sci. 24:6827. doi: 10.3390/ijms24076827, 37047798 PMC10095267

[ref18] Kreilaus F. Przybyla M. Ittner L. Karl T. (2022). Cannabidiol (CBD) treatment improves spatial memory in 14-month-old female TAU58/2 transgenic mice. Behav. Brain Res. 425:113812. doi: 10.1016/j.bbr.2022.113812, 35202719

[ref19] Naeini S. E. Bhandari B. Hill B. Perez-Morales N. Rogers H. M. Khodadadi H. . (2025). Rethinking Alzheimer’s: harnessing cannabidiol to modulate IDO and cGAS pathways for neuroinflammation control. Immunology 12:2025. doi: 10.1523/ENEURO.0114-25.2025, 41052930 PMC12519923

[ref20] Nitzan K. Bentulila Z. Bregman-Yemini N. Ayalon N. David D. Break E. . (2026). Sex-dependent effects of ultra-low-dose-THC preventive treatment on neuroinflammation and cognitive decline in 5xFAD mice. Biol. Sex Differ. 17:20. doi: 10.1186/s13293-025-00815-3, 41484932 PMC12866039

[ref21] Nitzan K. Ellenbogen L. Bentulila Z. David D. Franko M. Break E. P. . (2022). An ultra-low dose of ∆9-tetrahydrocannabinol alleviates Alzheimer’s disease-related cognitive impairments and modulates TrkB receptor expression in a 5XFAD mouse model. Int. J. Mol. Sci. 23:9449. doi: 10.3390/ijms23169449, 36012711 PMC9408848

[ref22] Palmieri B. Vadalà M. (2022). Oral THC: CBD cannabis extract in main symptoms of Alzheimer disease: agitation and weight loss. Clin. Ter. 174, 53–60. doi: 10.21203/rs.3.rs-1672277/v1 36655645

[ref23] Passmore M. J. (2008). The cannabinoid receptor agonist nabilone for the treatment of dementia-related agitation. Int. J. Geriatr. Psychiatry 23, 116–117. doi: 10.1002/gps.1828, 18081000

[ref24] Pertwee R. G. (2006). Cannabinoid pharmacology: the first 66 years. Br. J. Pharmacol. 147, S163–S171. doi: 10.1038/sj.bjp.0706406, 16402100 PMC1760722

[ref25] Raïch I. Lillo J. Rebassa J. B. Griñán-Ferré C. Bellver-Sanchis A. Reyes-Resina I. . (2025). Cannabidiol as a multifaceted therapeutic agent: mitigating Alzheimer’s disease pathology and enhancing cognitive function. Alzheimer’s Res. Ther. 17:109. doi: 10.1186/s13195-025-01756-0, 40394655 PMC12090481

[ref26] Ramírez B. G. Blázquez C. Del Pulgar T. G. Guzmán M. De Ceballos M. L. (2005). Prevention of Alzheimer’s disease pathology by cannabinoids: neuroprotection mediated by blockade of microglial activation. J. Neurosci. 25, 1904–1913. doi: 10.1523/JNEUROSCI.4540-04.2005, 15728830 PMC6726060

[ref27] Robson P. (2005). Human studies of cannabinoids and medicinal cannabis. Handb. Exp. Pharmacol. 168, 719–756. doi: 10.1007/3-540-26573-2_25 16596794

[ref28] Rosenberg P. B. Amjad H. Burhanullah H. Nowrangi M. Vandrey R. Pierre M. J. . (2026). A randomized controlled trial of the safety and efficacy of dronabinol for agitation in Alzheimer’s disease. Am. J. Geriatr. Psychiatry 34, 167–179. doi: 10.1016/j.jagp.2025.10.011, 41350162 PMC12681754

[ref29] Sánchez-Fernández N. Gómez-Acero L. Castañé A. Adell A. Campa L. Brito V. . (2024). A combination of Δ9-tetrahydrocannabinol and cannabidiol modulates glutamate dynamics in the hippocampus of an animal model of Alzheimer’s disease 21, e00439. doi: 10.1016/j.neurot.2024.e00439, 39232876 PMC11581878

[ref30] Volicer L. Stelly M. Morris J. McLaughlin J. Volicer B. J. (1997). Effects of dronabinol on anorexia and disturbed behavior in patients with Alzheimer’s disease. Int. J. Geriatr. Psychiatry 12, 913–919. doi: 10.1002/(SICI)1099-1166(199709)12:9<913::AID-GPS663>3.0.CO;2-D, 9309469

[ref31] Walther S. Mahlberg R. Eichmann U. Kunz D. (2006). Delta-9-tetrahydrocannabinol for nighttime agitation in severe dementia. Psychopharmacology 185, 524–528. doi: 10.1007/s00213-006-0343-1, 16521031

[ref32] Wang Y. Hong Y. Yan J. Brown B. Lin X. Zhang X. . (2022). Low-Dose Delta-9-tetrahydrocannabinol as beneficial treatment for aged APP/PS1 mice. Int. J. Mol. Sci. 23:2757. doi: 10.3390/ijms23052757, 35269905 PMC8910894

[ref33] Xiao Y. Dai Y. Li L. Geng F. Xu Y. Wang J. . (2021). Tetrahydrocurcumin ameliorates Alzheimer’s pathological phenotypes by inhibition of microglial cell cycle arrest and apoptosis via Ras/ERK signaling. Biomed. Pharmacother. 139:111651. doi: 10.1016/j.biopha.2021.111651, 34243602

[ref34] Yang S. Du Y. Li Y. Tang Q. Zhang Y. Zhao X. (2024). Tyrosine phosphorylation and palmitoylation of TRPV2 ion channel tune microglial beta-amyloid peptide phagocytosis. J. Neuroinflammation 21:218. doi: 10.1186/s12974-024-03204-6, 39227967 PMC11370263

